# Impact of specialized fatigue and backhand smash on the ankle biomechanics of female badminton players

**DOI:** 10.1038/s41598-024-61141-z

**Published:** 2024-05-04

**Authors:** Zhanyang He, Gongju Liu, Bin Zhang, Binyong Ye, Houwei Zhu

**Affiliations:** 1 College of Physical Education and Health Sciences, Zhejiang Normal University, Jinhua, China; 2 Scientific Research Center and Laboratory of Aquatic Sports Science of General Administration of Sports China, Zhejiang College of Sports, Hangzhou, China; 3 School of Competitive Sports, Beijing Sport University, Beijing, China

**Keywords:** Ankle biomechanics, Specialized fatigue, Badminton, Single leg landing, Jump smash, Limb non-dominance, Musculoskeletal system, Orthopaedics, Computer modelling, Biomedical engineering

## Abstract

During fatigued conditions, badminton players may experience adverse effects on their ankle joints during smash landings. In addition, the risk of ankle injury may vary with different landing strategies. This study aimed to investigate the influence of sport-specific fatigue factors and two backhand smash actions on ankle biomechanical indices. Thirteen female badminton players (age: 21.2 ± 1.9 years; height: 167.1 ± 4.1 cm; weight: 57.3 ± 5.1 kg; BMI: 20.54 ± 1.57 kg/m^2^) participated in this study. An 8-camera Vicon motion capture system and three Kistler force platforms were used to collect kinematic and kinetic data before and after fatigue for backhand rear-court jump smash (BRJS) and backhand lateral jump smash (BLJS). A 2 × 2 repeated measures analysis of variance was employed to analyze the effects of these smash landing actions and fatigue factors on ankle biomechanical parameters. Fatigue significantly affected the ankle-joint plantarflexion and inversion angles at the initial contact (IC) phase (p < 0.05), with both angles increasing substantially post-fatigue. From a kinetic perspective, fatigue considerably influenced the peak plantarflexion and peak inversion moments at the ankle joint, which resulted in a decrease the former and an increase in the latter after fatigue. The two smash landing actions demonstrated different landing strategies, and significant main effects were observed on the ankle plantarflexion angle, inversion angle, peak dorsiflexion/plantarflexion moment, peak inversion/eversion moment, and peak internal rotation moment (p < 0.05). The BLJS landing had a much greater landing inversion angle, peak inversion moment, and peak internal rotation moment compared with BRJS landing. The interaction effects of fatigue and smash actions significantly affected the muscle force of the peroneus longus (PL), with a more pronounced decrease in the force of the PL muscle post-fatigue in the BLJS action(post-hoc < 0.05). This study demonstrated that fatigue and smash actions, specifically BRJS and BLJS, significantly affect ankle biomechanical parameters. After fatigue, both actions showed a notable increase in IC plantarflexion and inversion angles and peak inversion moments, which may elevate the risk of lateral ankle sprains. Compared with BRJS, BLJS poses a higher risk of lateral ankle sprains after fatigue.

## Introduction

Badminton has gained a spot as one of the most popular sports globally^1^, with over 200 million participants worldwide^2^. This sport is often perceived to have a relatively safe non-contact condition^3^, but its injury risks are notably higher than what are commonly believed. The 2012 London Olympics statistics indicates that the injury rate of among badminton players is 15.9%, which is higher than the rates for other sports, such as tennis (11.4%), table tennis (6.3%), basketball (11.1%) and boxing (9.2%)^4^. Badminton ranks high in terms of injury rates. During play, badminton athletes perform various technical movements, including running, jumping, abrupt stopping, and lunging^5^. The frequent use of the lower limbs often results in a large proportion of lower limb injuries (58.0%–87.5% among professional players), with ankle injuries being the most prevalent^6–9^. In badminton matches, single-leg landings occur frequently in landing movements and account for approximately 21.07% of all landing actions (520 out of 2468 landings)^10^. The ankle joint, being the first joint to make contact with the ground, plays a major role in absorbing the impact of landings^11,12^. The prevalent pattern of single leg landings in badminton matches contributes significantly to the high frequency of ankle injuries, especially ankle sprains^13,14^.

**Figure 1:**
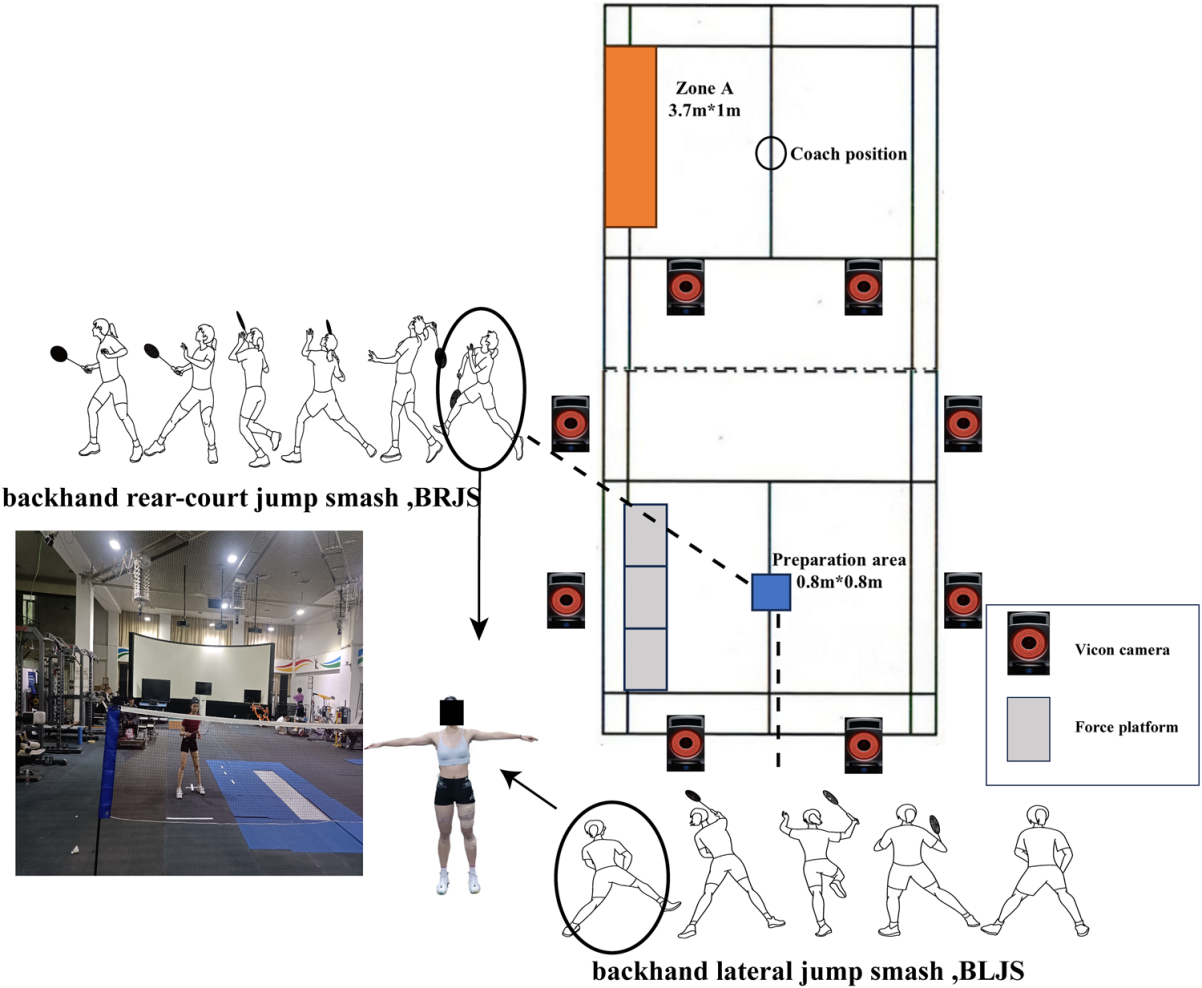
Experimental design and description of the smash landing action.

Jump smash in badminton is an important part of a player’s offensive strategy and accounts for 53.9% of scoring methods^15^. The majority of landings after a jump smash are on a single foot, with the contralateral foot touching the ground^16^. This type of landing movement places a heavy load on the joints of the unilateral lower limb^16^. Related studies have indicated that the risk of lower limb injury posed by backhand jump smashes (landing on the non-dominant foot) is a greater than the risk posed by forehand jump smashes (landing on the dominant foot), and injuries from this type of landing account for 47.6% of all single-leg landing injuries^17^. Backhand rear-court jump smash (BRJS) and backhand lateral jump smash (BLJS) are two common types of backhand jump smashes, with backward and lateral landing movements are the key focuses in preventing lateral ankle sprains in badminton^18^. In BRJS, using the right hand to hold the racket, the player needs to move laterally to an appropriate striking point and jump off the right foot to perform the smash. The player transfers his body weight from the back to the front then lands on the leg opposite to the racket-holding hand (non-dominant side) to maintain balance and push the torso from the back to the front (Fig. 1)^19,20^. Players always hold the racket with their dominant hand, and this limits their arm position when they strike in the backhand area. Combined with the inherent relationship between the dominant and non-dominant sides of the bilateral lower limbs, this condition inevitably creates an asymmetrical posture to balance the body^20^. In BLJS, using the right hand to hold the racket, the player quickly starts to catch an appropriate striking point, jumps off both feet with a lateral movement, and uses the rotation, bending, and extension of the torso to reach the optimal striking position and perform an aerial smash. The landing is still on the left foot (non-dominant side, Fig. 1)^21^. Previous studies have found that compared with players performing forehand lateral jump smashes, players performing BLJS balance their bodies with a greater hip abduction posture, and this non-dominant side landing strategy increases the load on the lower limb joints^10,22^. Furthermore, previous research has shown that in ankle injuries caused by non-dominant side landing movements, due to gender differences in neuromuscular control, women are more likely to suffer from ankle sprains than men during non-dominant side single-foot landings^23,24^. Therefore, the biomechanical mechanism of non-dominant side landing injuries in female athletes requires further attention.

The incidence of injuries during the final third of training and matches is linked to alterations in neuromuscular control due to fatigue^25–27^. Herbaut^28^ observed frequent ankle sprains, which are likely a consequence of fatigue, in the latter half of the season or during the second half of matches. Muscle fatigue adversely influences joint proprioception and the coordination and precision of movement control^29,30^. Therefore, fatigue is an important factor that affects the performance of athletes and alters the biomechanics of the lower limbs^31^. However, current fatigue protocols, especially those based on sport-specific movement patterns, seldom relate muscle fatigue experienced in badminton to biomechanical changes^32,33^.

This study was designed to analyze the influence of fatigue on ankle biomechanics during the landing phase among female badminton players performing two specific strokes: BRJS and BLJS. The hypothesis of this study is that fatigue will lead to increased ankle plantar flexion at initial contact and inversion angles at initial contact, as well as increased peak inversion moment, in female badminton players during the execution of BRJS and BLJS. An interaction effect may exist between the smashing movements and fatigue factors, that is, the changes in the simulated muscle strength of the ankle evertor muscle group under the influence of fatigue factors may differ between BRJS and BLJS movements.

## Methods

### Participants

Utilizing the G*Power 3.1 calculation with an alpha value of 0.05, a power value of 0.80, and an effect size of 0.40, the study required a minimum of 13 participants. Exactly 13 elite female badminton players from Beijing, China were recruited (age: 21.2 ± 1.9 years; height: 167.1 ± 4.1 cm; weight: 57.3 ± 5.1 kg; BMI: 20.54 ± 1.57 kg/m^2^), and they voluntarily participated in the study. The inclusion criteria were as follows: nationally ranked badminton players in China, right handed, and no injuries in the trunk, lower, or upper limbs for at least one year. Each participant was briefed on the experimental procedures and provided a written informed consent regarding their participation in the research. This work received ethical approval from the Ethics Committee of Beijing Sport University (Ethical Approval Number: 2021179H) and adhered to the principles of the Declaration of Helsinki.

### Procedure

The experiment was divided into three segments: data collection for pre-fatigue badminton-specific movements (Pre), implementation of a fatigue induction protocol, and data collection for post-fatigue badminton-specific movements (Post). The post-test was conducted within 5 min of the completion of the fatigue protocol^32^. A senior badminton coach served the shuttlecocks on the basis of each player’s striking height from a designated serving position. Before the experiment, the coach practiced the serving action in accordance with the player’s standing position height, the height of the upper limbs when stretched upward, and the player’s vertical jumping height to ensure the stability of the serving action. The shuttlecock serving height in badminton is equal to the distance from the ground when the athlete stands upright and stretches both upper limbs upward, which is 45–55% of the maximum vertical jumping height, plus the length of the badminton racket’s central axis^34^. Another coach was present outside the court at the start of the experiment to assess the quality of the athletes’ technical movements.

The experiment was conducted at the Biomechanics Laboratory of Beijing Sport University. Before the study, the participants were briefed on the steps and actions of the experiment and signed informed consent forms. Prior to the start of the experiment, the participants were asked to warm up on a treadmill at 8 km/h for 6 min, followed by 3 min of static stretching. Verbal encouragement was given before each test to ensure maximum effort from the athletes. Data collection started with the BRJS task followed by the BLJS task, and after each action, the players needed to return to the preparation area; the interval between actions was 20–30 s. The coaches and athletes considered this interval to be close to the pace of a real match. Three valid data sets were collected for each action. An action was considered valid when it met three conditions. First, the landing point of the shuttlecock after the jump smash must be in Zone A (3.7 × 1.0 m^2^, Fig. 1). Second, a pass motion quality assessment should be performed by off-field coaches. Third, the kinematic and kinetic data of the landing action must be completely collected by infrared high-speed cameras (Vicon) and force plates. The reason for the non-random order of action collection is that the jumping smash technique is usually adopted in an environment where opponents use high and deep shots, requiring athletes to make quick decisions about their action^35^. Therefore, in the experiment, we did not use a random order for data collection.

### Fatigue protocol

The fatigue protocol in this study was modified by a national-level coach on the basis of the Badminton-specific Speed Test (BST) developed and validated by Madsen et al.^36^. The modifications increased the randomness of the technical movements used to make the test protocol increasingly similar to actual scenarios where athletes experience fatigue during a match. The modifications resulted in a fatigue induction protocol that is close to actual match conditions. The participants started the fatigue protocol from a central pivot point. The athletes entered the agility area received a monitor that issued random commands, and were directed to touch the designated markers by using standard badminton footwork patterns as quickly as possible. After touching a marker, the athletes swiftly returned to the center of the court to perform the next command. All movements were conducted in accordance with basic sport-specific steps to ensure completion speed, with a metronome set before each participant’s test to dictate the pace. In this setting, the participants could complete the movements as immediately as possible within the specified amplitude. The Participants wore heart rate monitors throughout the execution of the fatigue model. The protocol consisted of 15 repetitions per set, with 15 s rest intervals between sets. The rate of perceived exertion (RPE) scale was used to assess the participants’ fatigue levels. Blood lactate levels and heart rate, were measured during rest intervals starting from the end of the third set. Participant who could not maintain the pace set by the metronome were instructed to engage in continuous vertical jumps until less than 70% of their maximum vertical jump height had been reached. Fatigue onset was determined using RPE ≥ 18 and blood lactate levels ≥ 8 mmol/L.

### AnyBody simulation

The lower-limb musculoskeletal model was developed using the biomechanical simulation software AnyBody version 7.4 (AnyBody Technology, Aalborg, Denmark), which has 3D motion capture dynamics. This software has undergone multiple experimental validations and demonstrates high reliability and accuracy^37,38^.

**Figure 2:**
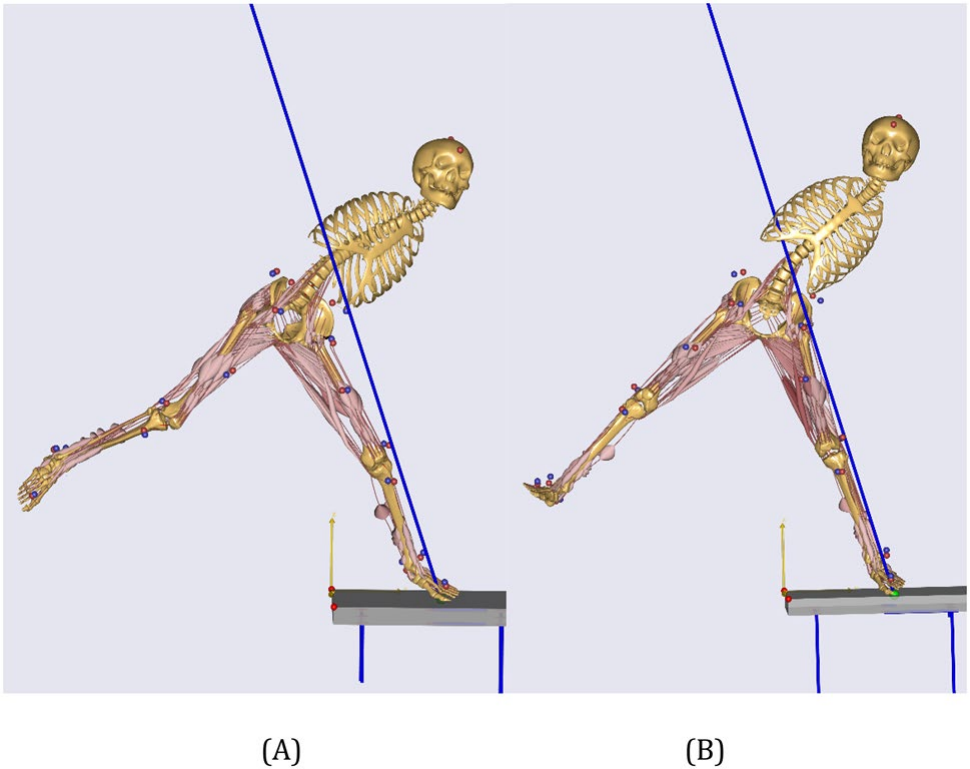
Bone-muscle models for two types of smash landing actions: (**A**) BRJS landing; (**B**) BLJS landing.

A standard multibody dynamics model was constructed in the AnyBody Modeling System (AMS), and it consisted of rigid components (e.g., human bones or external objects), kinematic actuators (e.g., bodily movements), and force/torque actuators (e.g., muscles). The forces and torques during motion were simulated using multibody dynamics simulation techniques. The AMS software contains over 1000 muscle elements^39^, which enable the detailed analysis of individual muscles, bones, and joints within the model. This analysis includes of those of forces, deformations, elastic properties of muscle tendons, antagonist muscle actions, and other biomechanically relevant characteristics^40,41^. This study utilized a lower-extremity bone-muscle model based on AMS, which employs Hill-type muscle models that comprise contractile, series elastic, and parallel elastic elements. Musculoskeletal models for the BRJS and BLJS were developed (Fig. 2). Optimization algorithms addressed muscle synergy issues within the skeletal muscle model to further enhance the validity of model data. The objective was the comprehensive scientific analysis of BRJS and BLJS.

### Collection and processing

The motion capture system comprised eight T40 Vicon cameras (Motion Analysis Raptor-4, the USA) operated at a sampling rate of 200 Hz and primarily captured kinematic parameters of the hip, knee, and ankle joints during smash movements. The 3D ground reaction forces (GRFs) were measured using three Kistler force plates and model 9287B (90 cm × 60 cm × 10 cm, Kistler Instruments AGCorp., Switzerland) at a sampling frequency of 1000 Hz. Synchronization of the motion capture system and force plates was ensured, and reflective markers (14 mm diameter) were placed on the participants’ anatomical landmarks in accordance with the Helen Hayes marker placement protocol. Segmental kinematics were captured using 24 retroreflective markers attached to the pelvis, lower limbs, and shoes of the participants^42^.

The analysis involved the kinematic and kinetic data from the Vicon Nexus software inputted into AnyBody 7.4 as C3D files. Musculoskeletal simulation models for BRJS and BLJS were established. The computational process was conducted in the following this sequence: reflective marker optimization, dynamic calculation, and inverse dynamics analysis^43^.

The analysis range was from the moment of ground contact to the maximum dorsiflexion of the ankle joint. Initial contact (IC) was defined as vertical GRF (VGRF) ≥ 10 N. The variables analyzed in this study for both movements included ankle-joint angles at IC at pre- and post-fatigue, ankle-joint range of motion (ROM) at pre- and post-fatigue, peak ankle-joint moments at pre- and post-fatigue, and peak simulated muscle forces at pre- and post-fatigue^18,32^.

### Statistical analysis

Kinetic data were normalized to the athlete’s body weight (dynamic parameter/body weight) using MATLAB R2019a (MathWorks, Natick, MA, the United States).

The normality of all variables was tested using the Shapiro–Wilk test, and the results indicated that the experimental data were normally distributed. Statistical analyses were conducted using SPSS 17.0 (SPSS Inc., Chicago, IL, the United States). A repeated-measures analysis of variance (2 × 2, smash action × fatigue factor) was employed to analyze the effects of smash actions and fatigue factors on the ankle biomechanical parameters. Post-hoc comparisons were conducted using paired t-tests with Bonferroni correction in cases with significant main effects. Data were presented as mean ± standard deviation, and statistical significance was considered at p-value < 0.05.

### Ethics statement

The studies involving human participants were reviewed and approved by Beijing Sport University ethics committee (Ethical Approval Number: 2021179H). The participants provided their written informed consent to participate in this study.

## Results

This study’s findings reveal that in the IC phase (Table 1), no significant interaction effect occurred between fatigue factors and smash actions on the IC angle of the ankle joint. Fatigue and smash action independently and significantly affected the plantar flexion angle (fatigue F(1,38) = 44.88, p < 0.001, action F(1,38) = 8.96, p = 0.005) and inversion angle (fatigue F(1,38) = 10.75, p = 0.002, action F(1,38) = 17.69, p < 0.001) of the ankle joint at IC (p < 0.05). The post-fatigue group demonstrated increased plantarflexion and inversion angles, with notable differences between the two smash actions. The effect of smash actions was significant only in the dorsiflexion/plantarflexion ROM (F(1,38) = 10.46, p = 0.003), with BLJS showing an overall greater dorsiflexion/plantarflexion ROM than BRJS.

**Table 1:** Comparison of the mean and standard deviation of biomechanical parameters for BRJS and BLJS pre and post-fatigue.

Variables	BRJS		BLJS		p-value		
	Pre	Post	Pre	Post	Fatigue	Action	Interaction
Initial contact (°)							
Dorsi-flexion (+)	− 22.45^ab^ (4.99)	− 27.42^ab^ (6.37)	− 20.51^ab^ (4.53)	− 24.46^ab^ (3.60)	** < 0.001**	**0.005**	0.223
Inversion (+)	− 1.02^ab^ (9.84)	3.80^ab^ (9.33)	5.18^ab^ (11.89)	9.56^ab^ (8.33)	**0.002**	** < 0.001**	0.888
Range of motion (°)							
Dorsi-flexion (+)	46.30^a^ (12.31)	46.58^a^ (13.56)	41.53^a^ (11.42)	40.63^a^ (10.55)	0.822	**0.003**	0.753
Inversion (+)	16.71 (12.58)	13.33 (10.4)	13.52 (10.36)	15.40 (10.85)	0.640	0.728	0.150
Peak ankle moment (Nm/kg)							
Dorsi-flexion (+)	− 2.09^ab^ (0.34)	− 1.94^ab^ (0.41)	− 1.84^ab^ (0.27)	− 1.68^ab^ (0.36)	** < 0.001**	** < 0.001**	0.953
Inversion (+)	1.15^ab^ (0.21)	1.34^ab^ (0.12)	1.24^ab^ (0.19)	1.42^ab^ (0.19)	** < 0.001**	**0.006**	0.769
Internal (+)	0.74^a^ (0.25)	0.75^a^ (0.26)	0.93^a^ (0.29)	1.01^a^ (0.28)	0.258	** < 0.001**	0.252
Peak muscle strength (N/kg)							
SOL	25.90 (1.40)	25.64 (2.53)	25.96 (1.10)	25.85 (2.13)	0.548	0.666	0.652
GL	29.01^ab^ (1.47)	28.00^b^ (2.69)	27.91^a^ (1.90)	27.46 (2.01)	**0.048**	**0.014**	0.387
GM	51.86^a^ (6.08)	49.79^a^ (8.28)	47.99^a^ (5.19)	46.06^a^ (5.92)	**0.041**	**0.001**	0.942
TP	7.52 (1.13)	7.40^a^ (1.46)	7.47^b^ (0.75)	6.85^ab^ (1.62)	0.105	0.080	0.078
TA	9.01^b^ (2.21)	7.70^ab^ (1.61)	9.34 (1.65)	9.12^a^ (2.66)	**0.023**	**0.005**	0.062
PB	8.63^a^ (0.74)	8.53^a^ (0.75)	8.99^a^ (0.44)	9.05^a^ (0.65)	0.819	** < 0.001**	0.483
PL	11.79^a^ (0.73)	11.63^a^ (0.59)	12.44^ab^ (0.40)	11.94^ab^ (0.53)	**0.004**	** < 0.001**	**0.037**

*SOL* soleus, *GL* gastrocnemius lateralis, *GM* gastrocnemius medialis, *TP* tibialis posterior, *TA* tibialis anterior, *PB* peroneus brevis, *PL* peroneus longus.

Significant values are given in bold.

^a^Statistically significiant difference compared with other action (< 0.05).

^b^Statistically significiant difference between pre-fatigue and post-fatigue (< 0.05).

Kinematically, the effect of the interaction between fatigue and smash actions on the peak moments of the ankle joint was not significant. However, the type of smash actions exerted a significant influence on the peak ankle moments, with BRJS revealing greater plantarflexion moment and reduced inversion and internal rotation moments. Fatigue significantly affected the peak dorsiflexion/plantarflexion (F(1,38) = 15.61, p < 0.001) and peak inversion/eversion (F(1,38) = 50.13, p < 0.001) moments, resulting in their post-fatigue decrements.

Observation of the simulated muscle force dynamics revealed the significant influence of the interaction between smash actions and fatigue factors on the force of the peroneus longus (PL) (F(1,38) = 4.68, p = 0.037), with notable differences in PL force between the two smash actions and a significant decrease in PL force due to fatigue in the BLJS task. The fatigue factors also significantly affected the lateral gastrocnemius (GL) (F(1,38) = 4.18, p = 0.048), medial gastrocnemius (GM) (F(1,38) = 4.47, p = 0.041), and tibialis anterior (TA) (F(1,38) = 5.64, p = 0.023), led to their reduced muscle forces. The smash actions exhibited significant effects on GL (F(1,38) = 6.64, p = 0.014), GM (F(1,38) = 11.93, p = 0.001), TA (F(1,38) = 8.73, p = 0.005), and peroneus brevis (PB) (F(1,38) = 16.68, p < 0.001), In the BLJS task, the muscle forces of GL (gastrocnemius lateralis) and GM were lower than those in the BRJS task, while the muscle forces of TA and PB in the BLJS action were greater than those in BRJS.

## Discussion

A musculoskeletal modeling approach was utilized in this study to investigate ankle-joint biomechanical differences during two specific backhand smash landing actions in badminton in the context of sport-specific fatigue. The findings reveal distinct landing strategies employed during BRJS and BLJS, with the fatigue factors influencing the ankle-joint biomechanical parameters. Consistent with the initial hypothesis, the ankle dorsiflexion angles at IC and the inversion angles at IC for BRJS and BLJS increased after badminton-specific fatigue intervention, and the peak inversion moment increased. Our hypothesis regarding the different effects of fatigue on the simulated muscle strength of the ankle evertor muscle group during the two movements was partially validated. The effect of the interaction between the smashing movements and fatigue factors on the muscle force of the PL was significant. The post-hoc tests revealed a significant reduction in the peak muscle force of PL in the BLJS group, but no significant difference was found in the BRJS group. No interaction effect was observed on the peak muscle force of the PB.

Badminton smash is a common scoring technique^43^, and understanding of the effect of fatigue on the biomechanical parameters of ankle joint during smash landing actions is crucial to obtaining insights into its biomechanical effects during single-leg landing. Previous studies have revealed the negative effect of fatigue on single-leg landing actions in smashes^31,45^. Therefore, this study collected kinematic and kinetic data on the ankle joint during single-leg landings during BRJS and BLJS. Fatigue exerted a considerable influence on the kinematics of ankle-joint contact during landing. A previous study on male badminton players reported a decreased plantarflexion angle at the ankle joint during forehand smash single-foot landings, a phenomenon that was also observed in forefoot runners^32,46^. However, our findings indicate a substantial increase in the plantarflexion angle at the ankle-joint contact, a result that contradicts those of previous research. This discrepancy may be attributed to variations in the side of smash execution (dominant versus nondominant) and gender-related landing differences. Similar studies have identified variations in ankle-joint kinematics between forehand and backhand smash actions^47^, with females exhibiting a larger IC plantarflexion angle in single-leg landings compared with males^48^.

Fatigue leads to increased plantarflexion and inversion during ankle-joint landing^49,50^, which affects foot stability and position. An increase in the plantarflexion angle during landing can result in a lax foot position and decreased joint stability^51^. In the landing phase, the centripetal contraction of plantar flexors slows down the descent of the heel after foot contact^32^. Fatigue impairs neuromuscular recruitment capabilities^52^, leading to decrements in the peak plantarflexion moment and strength of the plantar flexors (GL and GM) in resisting GRFs on the sagittal plane. Our study also revealed remarkable fatigue effects on TA strength, indicating that badminton-specific fatigue affects muscle recruitment capabilities beyond the plantar flexors. Badminton involves various movement patterns that contribute to varying degrees of fatigue across different muscle groups^53,54^.

This study also examined the biomechanical parameters of ankle joint during two badminton smash actions: BRJS and BLJS landings. The different landing directions during these smashes enable the execution of varied ankle-joint landing strategies and the attainment of various biomechanical parameters^55^. Smash action showed a significant main effect in terms of the ankle joint’s contact angle and peak moment. Compared with BRJS, BLJS had a smaller plantarflexion angle and peak plantarflexion moment but a larger inversion angle, peak inversion moment, and peak internal rotation moment during contact. A previous study reported that the peak vertical GRF, peak inversion moment, and internal rotation moment during nondominant-side lateral landings are much greater than those during forward- and opposite-side landings^55^. Previous research explored ankle-joint biomechanics in forward, diagonal, and lateral single-leg landings but rarely focused on the biomechanical factors that influence rearward single-leg landings^56–58^.

The fatigue factors and smash action significantly influenced the force of PL. In the BLJS task, fatigue resulted in a statistically significant reduction in PL muscle force. Rodrigues^59^ observed that fatigue in PL led to a reduced contraction strength and subsequent ankle instability. The PL plays a crucial role in resisting the inversion moments of the foot or ankle complex and maintaining mediolateral stability^60,61^. Therefore, players executing BLJS may face a higher risk of ankle inversion injuries compared with those performing BRJS.

Prior studies established the increased likelihood of ankle sprain injuries observed resulting from landing with increased ankle plantarflexion and inversion angles post-fatigue^62–64^. Consistent with these findings, our results show a similar increase in plantarflexion and inversion angles at the ankle joint during landing under fatigue. Moreover, a notable elevation in peak inversion torque was detected post-fatigue, which further amplified the potential for ankle sprain risks.

Research describes lateral ankle sprains as typically caused by a combination of ankle inversion, plantarflexion, and internal rotation^64^. In this study, the BLJS task had a high landing inversion angle, peak inversion moment, and peak internal rotation moment. Increased peak inversion and internal rotation moments at the ankle can elevate the risk of sprains^65,66^. Hence, a higher risk of lateral ankle sprains is observed with lateral smash actions (e.g., BLJS). Furthermore, PL, which plays a key role in the prevention of ankle inversion, exhibited a significant interaction effect. The effect of fatigue on the muscle force of PL in the BLJS task was greater than that in the BRJS task, leading to a more pronounced decrease in the muscle force of PL in the lateral smashes (BLJS) compared with that in the rearward smashes (BRJS). An insufficient counteracting moment produced by PL during ankle inversion can lead to the loss of ankle control^67^. Therefore, given these kinematic, joint dynamic, and muscular force findings, the risk of ankle injury post-fatigue is greater in lateral smashes (BLJS) than in rearward smashes.

This study has certain limitations. First, although the use of musculoskeletal modeling to solve ankle muscle forces allowed us to understand the force situation of deep muscles, we did not examine actual muscle activity. Second, our study only investigated female athletes, so the current results may not be applicable to male populations. Future research, should include male athletes in their study scope and use high-density surface electromyography equipment to thoroughly understand the phenomenon of muscle fatigue.

## Conclusion

In this study, fatigue and smash actions, specifically BRJS and BLJS, considerably affected biomechanical changes in the ankle joint. After fatigue, both actions showed a notable increase in the IC plantarflexion and inversion angles and peak inversion moments, which led to an elevated risk of lateral ankle sprains. Compared with BRJS, the BLJS poses a greater risk of lateral ankle sprains post-fatigue. Athletes should minimize the use of BLJS during fatigue to reduce the incidence of injuries.

## Data Availability

The dataset supporting this article is available on request to the first corresponding author.
